# Perception of Doctors Attending Association of Black Psychiatrists-UK Led CASC Preparatory Course

**DOI:** 10.1192/bjo.2022.103

**Published:** 2022-06-20

**Authors:** Suraju Adeyemo, Olufikunayo Manuwa, Theresa Ugalahi, Nneamaka Asiodu, Sewanu Awhangansi, Babatunde Odebiyi, Nwife Akhator-Eneka, Mojisola Adeniji, Chukwuma Oraegbunam

**Affiliations:** 1 Lancashire and South Cumbria NHS Foundation Trust, Preston, United Kingdom; 2 Foward Thinking Birmingham NHS, Birmingham, United Kingdom; 3 Leeds Community Healthcare, Leeds, United Kingdom; 4 Black Country Healthcare NHS Foundation Trust, Dudley, United Kingdom; 5 Leicestershire Partnership NHS Trust, Leicester, United Kingdom; 6 Greater Manchester Mental Health NHS Foundation Trust, Manchester, United Kingdom; 7 Tees Esk and Wear Valley NHS Trust, Darlington, United Kingdom

## Abstract

**Aims:**

Within the United Kingdom, there has been differential attainment in academic performance and career progression between International Medical graduates (IMG) and UK medical school graduates. Reasons reported for these differences include; poor relationships with trainers, cultural differences, and inadequate support. To close this differential attainment, effective interventions to support IMGs are indicated. This need for diversity led to the creation of the Association of Black Psychiatrists-UK Tutorial group (The-Tutors). The Tutors is a free online CASC preparatory group that started about 2-years ago and is tailored to meet the specific needs of black doctors sitting the Royal College of Psychiatrists examination. over 100-black Doctors have benefited from the group. This study was conducted to evaluate the experience of these doctors.

**Methods:**

The sampling frame was the population of doctors who attended The-Tutors before sitting the CASC examination. Data were collected on socio-demographic characteristics, past psychiatry training experiences, and participants' experience toward preparation and passing CASC.

An online questionnaire was completed and this was distributed through social media (closed WhatsApp groups).

Responses were anonymous.

**Results:**

Total participants were 33, out of which 20 (60.6%) had passed the CASC examination while 13 (39.4%) are still awaiting results.

The majority (51.5%) of the participants had only international training in Psychiatry, 27.3% had their training in the UK, and 12.1% had both, while 9.1% had no formal-psychiatric training.

Most 21(63.6%) participants indicated that the group was ‘extremely useful’ for CASC preparation.

In terms of comparing experience in this group with other CASC preparatory groups, all the respondents found The Tutors group more helpful; 16(48.5%) indicated that the group was ‘extremely helpful’, 14(42.4%) ‘very helpful’ and 9.1% “moderately helpful”.

Most of the participants (75.8%) indicated that they were “extremely likely” to recommend the group to others.

The majority (>65%) of respondents reported that The-Tutors helped in improving their knowledge, communication skills, confidence approaching the examination, and motivation to study.

Close to half (48.5%) of participants who had passed the CASC examination indicated that The-Tutor was “extremely helpful” toward their success.

**Conclusion:**

This study has shown positive experiences of IMG especially of Black ethnic group attending an all- Black-led CASC preparatory group. This could be an indication that support groups specifically targeted toward the needs of IMG could help lead to an increase in success rates in UK examinations.

